# Supplementary material for: Expanding the Diversity of Bacterioplankton Isolates and Modeling Isolation Efficacy with Large-Scale Dilution-to-Extinction Cultivation
Source: Appl Environ Microbiol. 2020 Aug 18;86(17):e00943-20. doi: 10.1128/AEM.00943-20 (PMC7440811; doi:10.1128/AEM.00943-20)
Supplement: Supplemental file 1 [file AEM.00943-20-s0001.pdf]

Supplemental Information

Supplemental Text
Math for back calculating the per-cell division rates in Henson et al. 2018 assuming 15%--55% viability.

Denote the initial cell count as X_0, and the cell count in n^{th}generation asX_n. If all the cells are viable, per each generation, all the cells are doubling:

$$X_n = X_{n-1} \cdot 2 \quad \text{(eq. S1.1)}$$

And therefore, after $n$generations, the cell count $X_n$would be:

$$X_n = X_0 \cdot 2^n \quad \text{(eq. S1.2)}$$

When not all the cells are viable, for each generation, instead of simply doubling the whole population, only a portion of the cells (denoted as$V < 1$) replicates. In this case:

$$X_n = X_{n-1} \cdot (1 + V) \quad \text{(eq. S1.3)}$$

Here we can see that (eq. 3.1) is actually a special case of (eq. 3.3), when $V = 1$,

$$X_n = X_{n-1} \cdot (1 + 1) = X_{n-1} \cdot 2$$

When $V < 1$, after n generations:

$$X_n = X_0 \cdot (1 + V)^n \quad \text{(eq. S1.4)}$$

Denoting the total time to get n generations as t, which means the number of generations per unit time, also known as the per-cell division rate, is $\mu = n/t$. Rewriting (eq. 3.4), we have:

$$X_n = X_0 \cdot (1 + V)^{\mu \cdot t} = X_0 \cdot 2^{\mu \cdot log_2(1+V) \cdot t} \quad \text{(eq. S1.5)}$$

According to (eq. 3.5), a cell population of Vviability and μ per cell division rate, the population division rate is:

$$\mu_{population} = \mu \cdot log_2(1 + V) \quad \text{(eq. S1.6)}$$

Henson MW, Lanclos VC, Faircloth BC, Thrash JC. 2018. Cultivation and genomics of the first freshwater SAR11 (LD12) isolate. ISME J 12:1846–1860. Doi: 10.1038/s41396-018-0092-2 showed that the optimal population division rate of SAR11 LD12 is $\mu_{population} = 0.5\ day^{-1}$, assuming the viability of LD12 is $V = 0.15$, by plugging in these two numbers to (eq. S1.6), we can get the per-cell division rate of LD12 is:

$$\begin{aligned} \mu &= \mu_{population}/log_2(1 + V) \\ &= 0.5\ day^{-1}/log_2(1 + 0.15) \\ &= 0.5\ day^{-1}/0.20163 \\ &= 2.48\ day^{-1} \end{aligned}$$

If assuming $V = 0.55$, $\mu = 0.79\ day^{-1}$. Therefore, given the range of the viability of LD12 is between 15%--55%, the corresponding per cell division rate is between 0.79 divisions to 2.48 divisions per day.

Supplemental Table
Supplemental Table S1 is a spreadsheet, Table_S1.xlsx. This includes MWH media recipes, ASV and OTU tables, taxonomic and relative abundance information for all isolates, NMDS information, biogeochemical data for the samples, and the BLAST results of isolate hits. Available at https://doi.org/10.6084/m9.figshare.12142113.

Supplemental Figures follow, with captions embedded

Figure S1

16S rRNA gene phylogeny of LSUCC isolates in the Phylum Actinobacteria. Scale bar represents 0.01 changes per position. Values at internal nodes indicate bootstrap values (n = 1000).

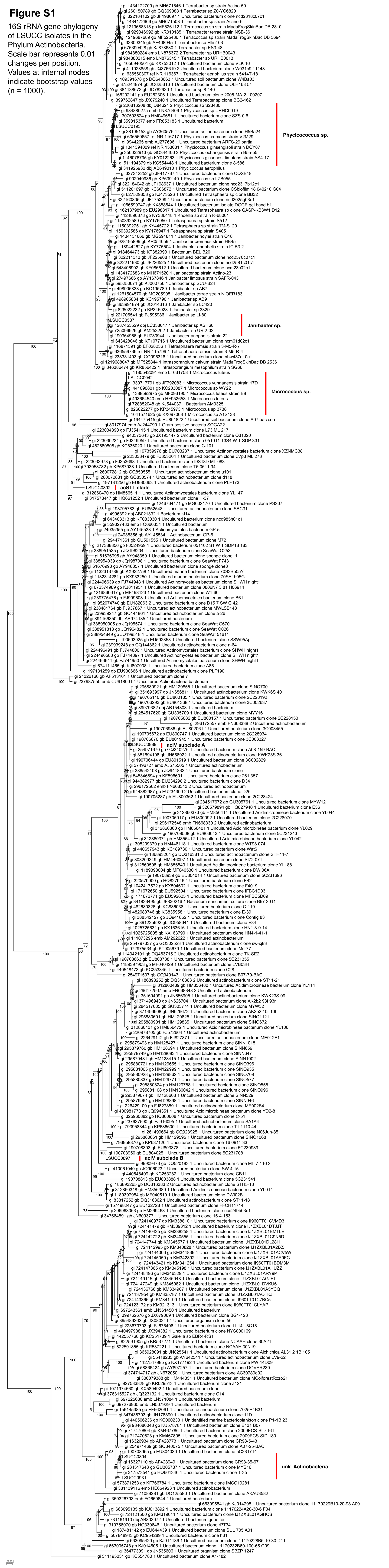

Figure S2

16S rRNA gene phylogeny of LSUCC isolates in the Class Alphaproteobacteria. Scale bar represents 0.01 changes per position. Values at internal nodes indicate bootstrap values (n = 1000).

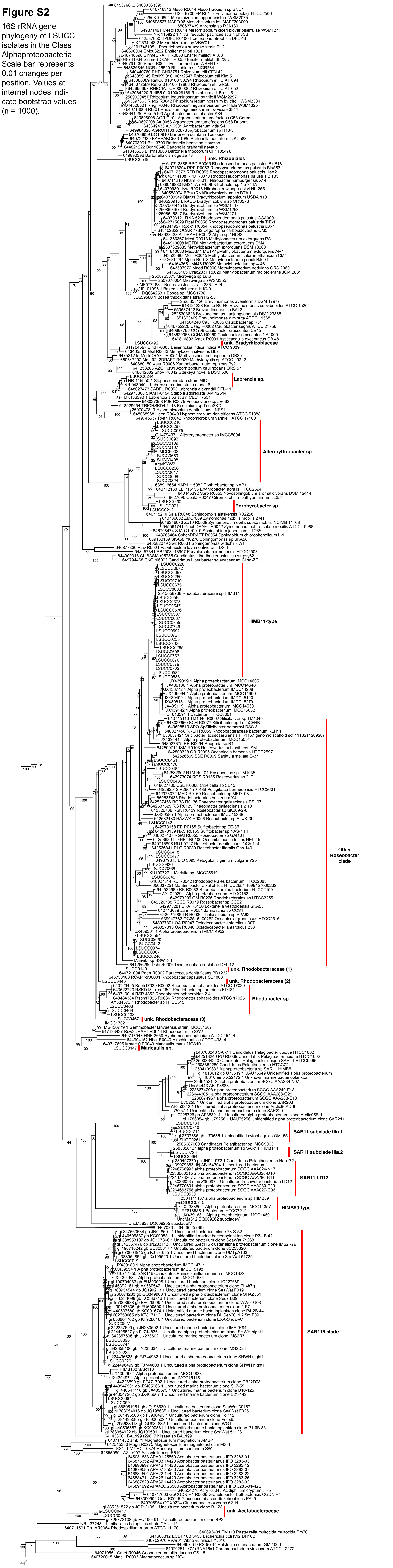

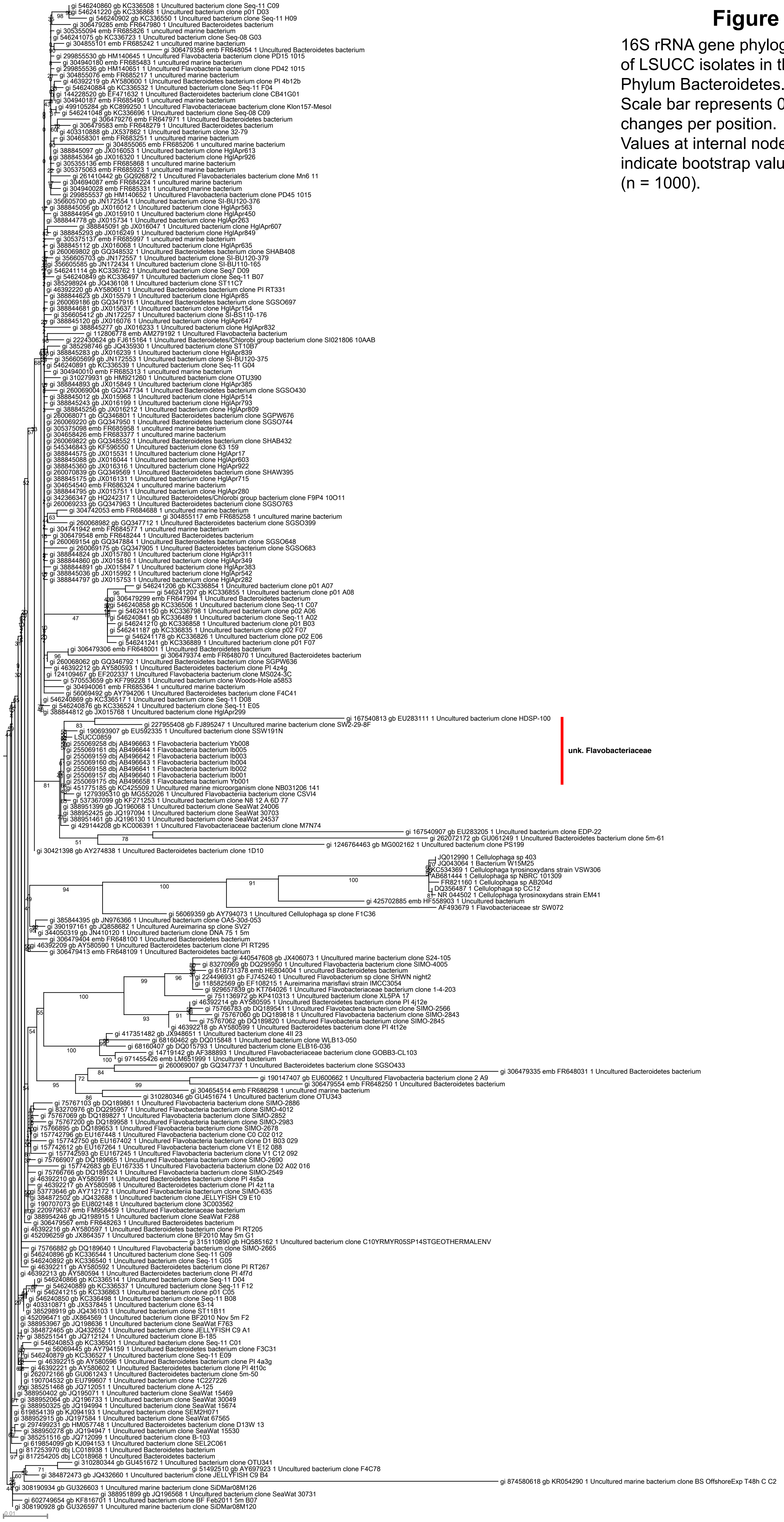

Figure S3

16S rRNA gene phylogeny of LSUCC isolates in the Phylum Bacteroidetes. Scale bar represents 0.01 changes per position. Values at internal nodes indicate bootstrap values (n = 1000).

Figure S4

16S rRNA gene phylogeny of LSUCC isolates in the Class Betaproteobacteria. Scale bar represents 0.01 changes per position. Values at internal nodes indicate bootstrap values (n = 1000).

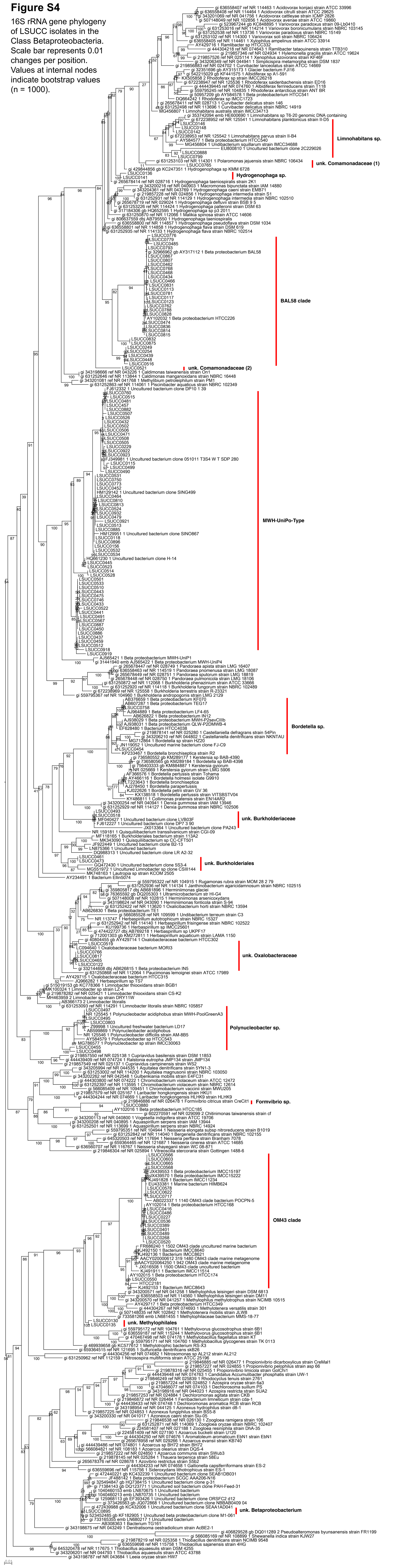

Figure S5

16S rRNA gene phylogeny of LSUCC isolates in the Class Gammaproteobacteria. Scale bar represents 0.01 changes per position. Values at internal nodes indicate bootstrap values (n = 1000).

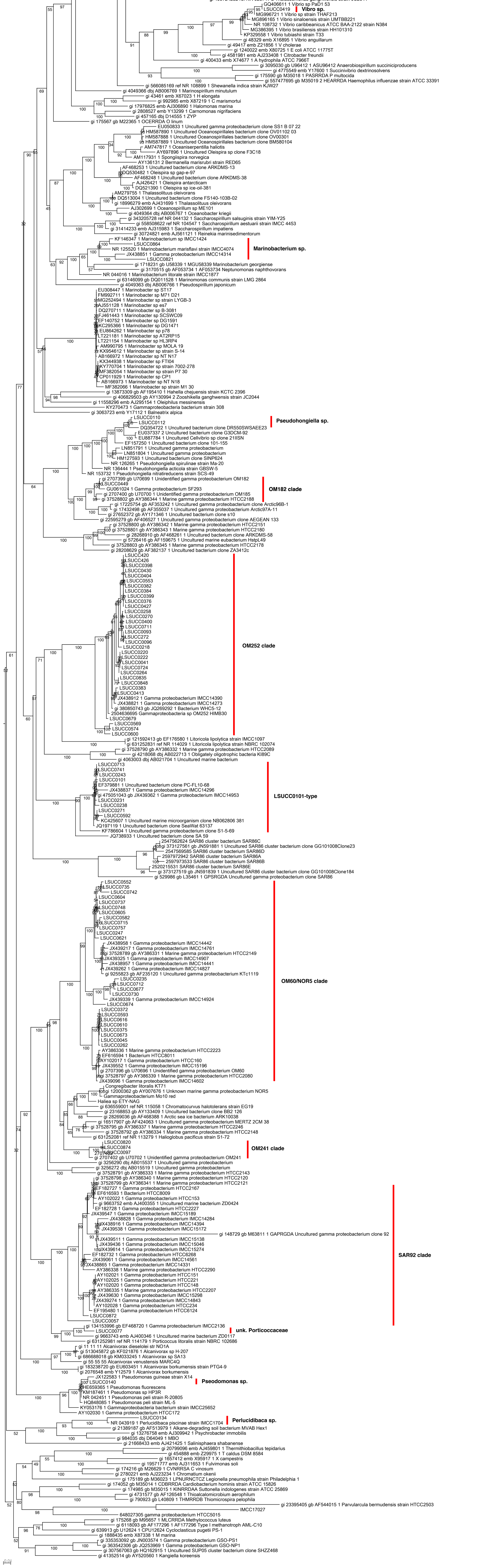

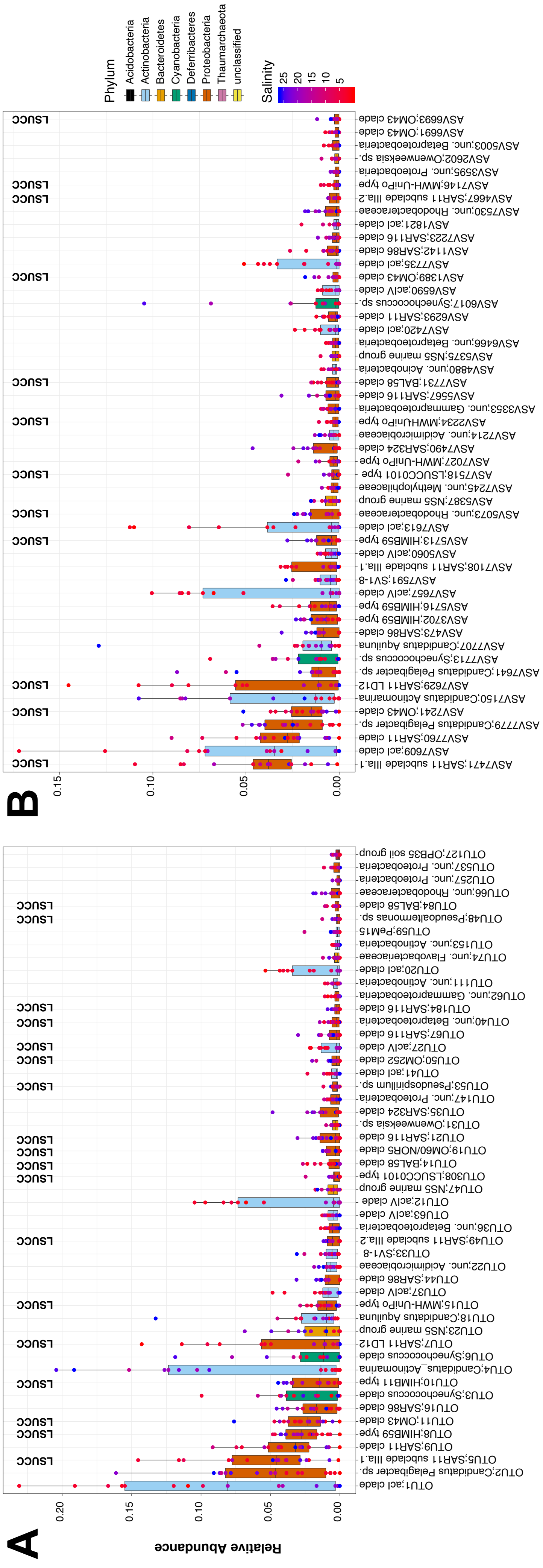

Figure S6. Rank abundance plot of the top 50 most abundant (A) OTUs and (B) ASVs across all sites. The boxes indicate the interquartile range (IQR) of the data, with vertical lines indicating the upper and lower extremes according to 1.5 x IQR. Horizontal lines within each box indicate the median. The underlying data points are each individual OTU's sample relative abundances. The shade of the dot represents the salinity, while the color of the box is the Phylum of the OTU. OTUs with cultured representatives from the LSU culture collection are labeled with one LSUCC isolate.

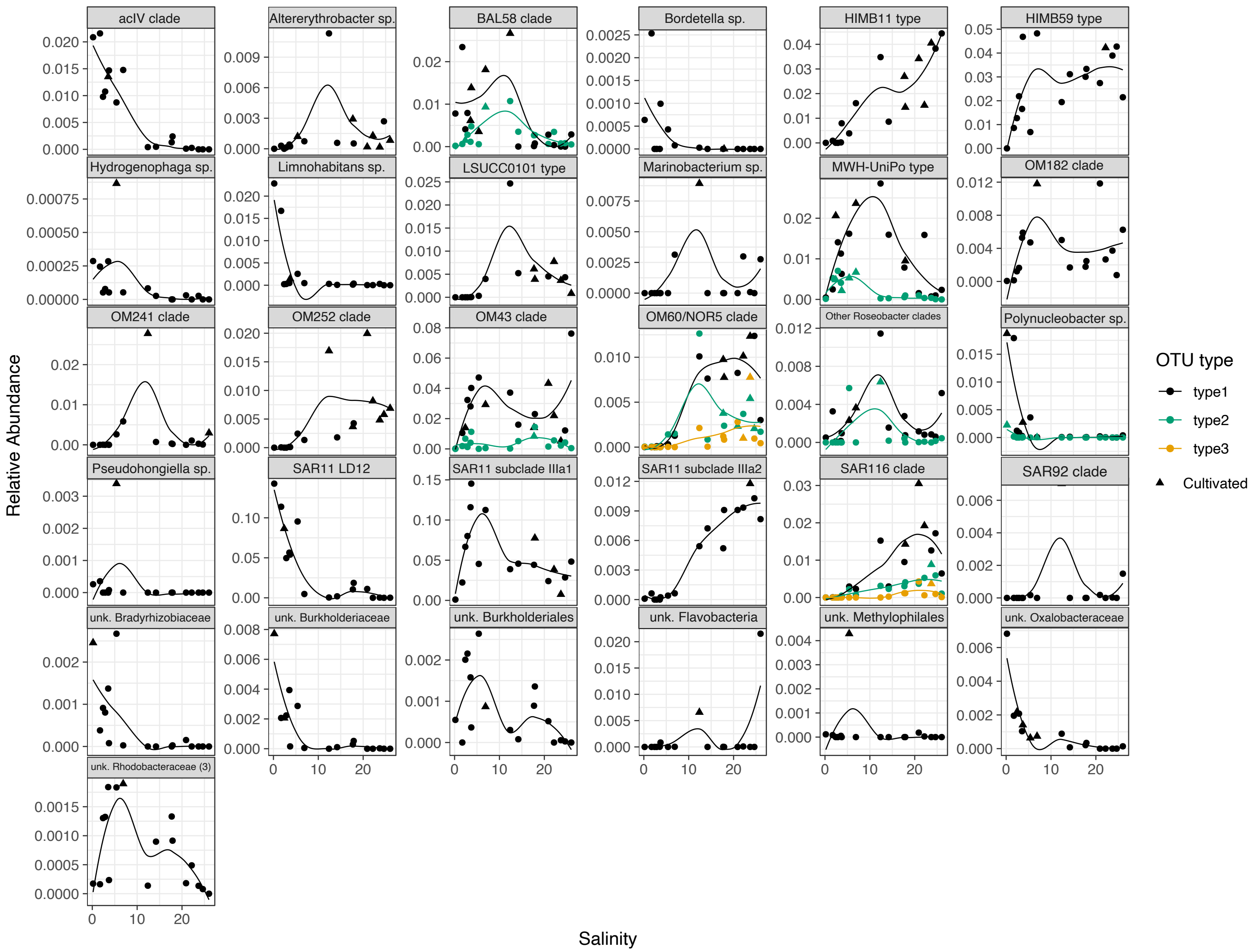

Figure S7. Relative abundance of cultivated OTUs according to site salinity. The color of the line represents the different OTUs classified within the LSUCC group. Non-linear regressions have been added for reference.

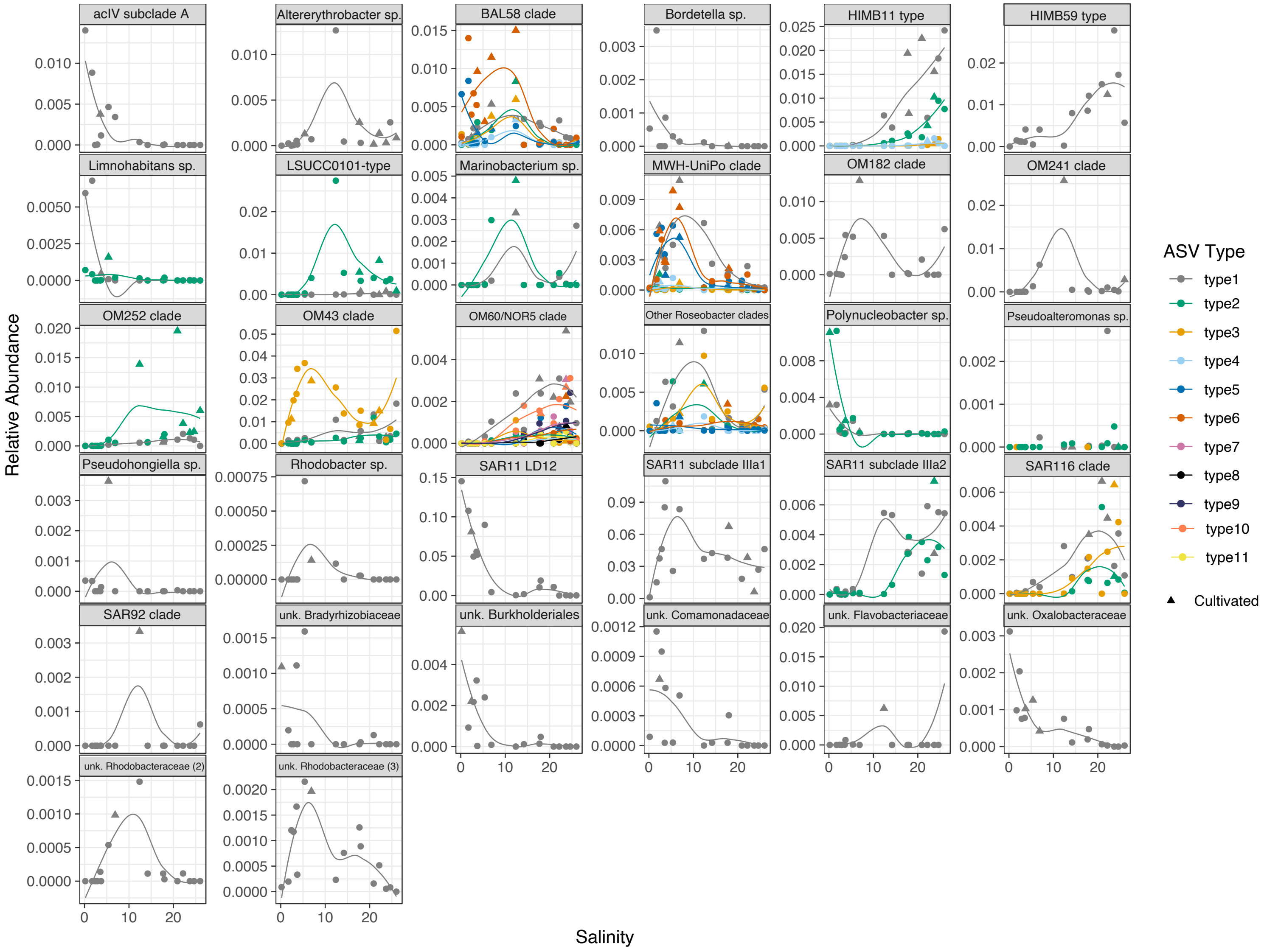

Figure S8. Relative abundance of ASVs with a cultured LSUCC representative according to site salinity. The color of the line represents the different ASVs classified within the LSUCC group. Non-linear regressions have been added for reference. Arrows represent sites where isolates were cultivated.

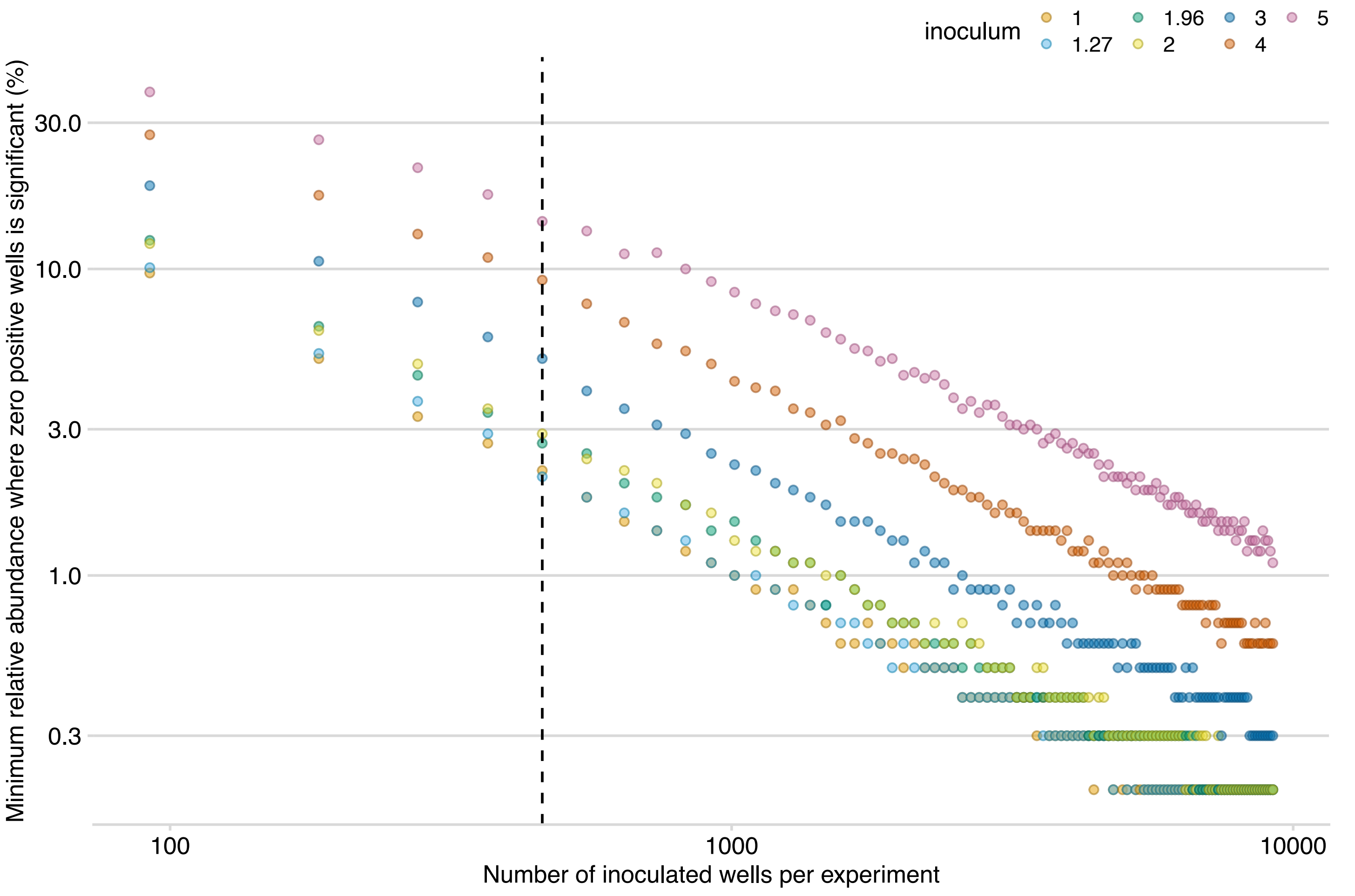

Figure S9. Sensitivity testing of the viability model to determine minimum numbers of wells required to overcome false negatives associated with differing levels of taxon relative abundance. Different inoculum densities (λ) are depicted with colors. The dotted line indicates 460 wells which corresponded to the experiments in this study.

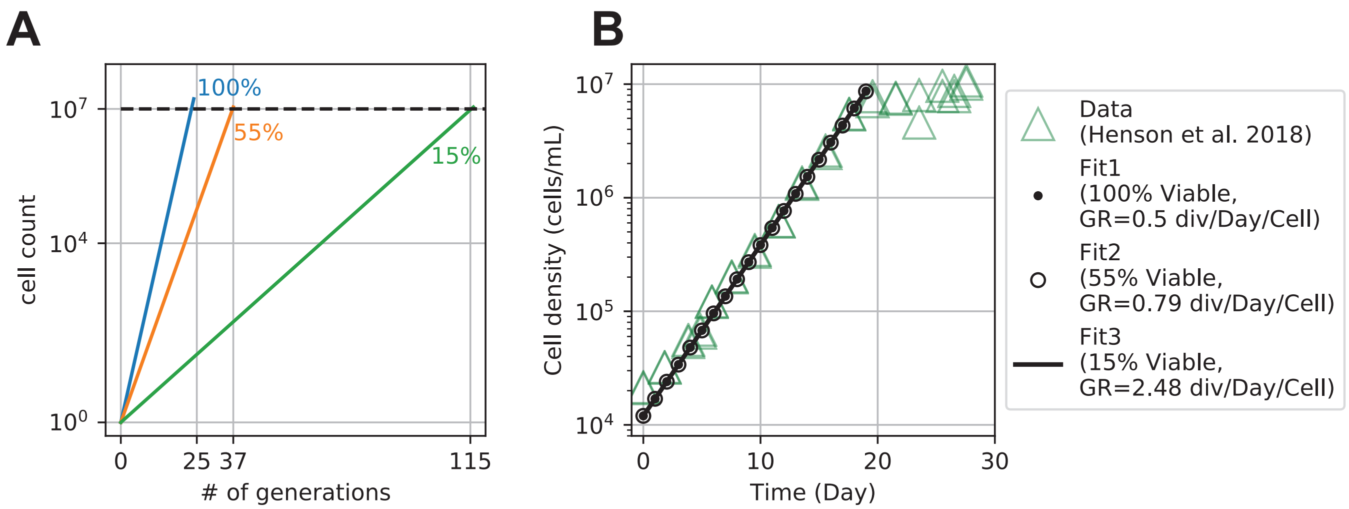

Figure S10. Logarithmic growth with different percentages of cell viability. Under different cell viabilities, such as 100%, 55% and 15% shown in (A), the culture can have logarithmic growth in the population size if we assume no cell death. It takes around 25 generations to have 10^7fold expansion of population size when all cells are active (100% viable), whereas when 15% of the population is actively duplicating, it takes around 115 generations to reach the same population size expansion. B) For the SAR11 LD12 strain LSUCC0530, the previously measured population doubling rate was 0.5/day (29). If all the cells are active (100% viability), each cell within the population will double 0.5 times per day (Fit 1 in B). However, with 15% viability, the actual active cell doubling rate is 2.48 doublings day-1 (Fit 3 in B). Henson et al 2018 full reference (from key): Henson MW, Lanclos VC, Faircloth BC, Thrash JC. 2018. Cultivation and genomics of the first freshwater SAR11 (LD12) isolate. ISME J 12:1846–1860. Doi: 10.1038/s41396-018-0092-2
